# Heating and Ventilation

**Published:** 1895-04-20

**Authors:** 


### PRACTICAL DEPARTMENTS.

HEATING AND VENTILATION—(*Continued from p.* 33).

Natural *versus* Artificial Ventilation.

Before leaving the subject of hospital heating and ventilation it may be well briefly to see how the weight of experience goes to show, from the facts gathered together by authorities on such matters, which systems have been found in actual practice to work the best. In doing so it must be borne in mind that in the treatment of such buildings as hospitals very many considerations have to be studied which do not enter into that of other houses and institutions.

Much has been said and written about the subject of late years since the vast importance of the matter has come to be more adequately realised, but the opinions, even of experts, still differ so widely that no very universal conclusion has even yet been arrived at.

In America and continental countries climatic exigencies render mechanical means of ventilation almost essential. When for many months in the year open windows are prohibited by the coldness of the external temperature, or where the stillness of the atmosphere prevents sufficient interchange of air currents, it is readily seen that some artificial means must be adopted to ensure the ventilation necessary for buildings holding within their walls a large number of sick people. In Mr. Burdett's "Hospitals and Asylums of the World" a great deal of information is to be found upon the various systems in vogue in the United States and other countries, too numerous to be touched upon here. Artificial ventilation can be effected either by "propulsion" or "extraction," the former system being, we believe, that which has yielded the best results. Both systems were tried at the Lariboisière Hospital, Paris, on the two different sides of the building. Comparison of results showed that while by the propulsion method the air supply was 3,178 cubic feet per head per hour, by the rival system the amount was only 1,059 cubic feet; the former, however, being more than twice as expensive as the latter.

In England the conditions of temperature are widely different. Open windows and free ventilation from outside are possible almost every day throughout the year, and experience has certainly so far gone to prove that the elaborate and expensive systems of mechanical ventilation and heating which may be necessary under certain conditions are not only unnecessary, but where applied have undoubtedly failed to yield results justifying their application. Many instances might be given in support of this view, but we will only quote one notable instance—that of the York County Hospital. This building was supplied with a combined system of heating and ventilation, which, says Mr. Burdett, "was exclusively relied on for keeping the ward air up to its proper standard of purity . . . the windows were simply glazed panels with no means of opening, and there were no open fire-places in the wards. This system continued in action for some nine years, during which time complaints were frequent of the close and offensive state of the ward air. Wound diseases prevailed and increased until at last it became impossible to make the smallest incision without erysipelas supervening. After urgent representations by the medical staff the hospital was at last closed, the artificial system abandoned, windows were made to open, fire-places were put into the wards, and every part was thoroughly cleansed. Since that time erysipelas as a hospital disease has disappeared, and operations do well."

Such failures as this no doubt in many cases are more due to inefficient working than the fault of the system as a system, and Sir Douglas Galton, in his "Hospital Construction," mentions that he has visited "on several different occasions" three important hospitals in Europe and the States where ventilation by propulsion was the established method, on each occasion it happening that the propulsion was out of use for the time; "in some cases evidently," adds the author, "with the object of saving the expense of fuel." And this danger of a system, good in itself if adequately maintained, being tampered with on grounds of economy by those ignorant of the force with which they have to deal, is perhaps one of its greatest objections. On this head Sir Douglas Galton's experience does not by any means stand alone. In a report of the Barracks and Hospital Improvement Commission, mention is specially made of a hospital "ventilated with one of the most perfect apparatus we have anywhere seen," professing to supply between 4,000 and 5,000 cubic feet of air per bed per hour, where the atmosphere of the wards was described as being utterly stagnant and foul, a condition caused by the tampering with one of the valves of the supply pipe, apparently for no other reason but "to save fuel by diminishing the quantity of warm air supplied to the sick."

An experiment of this kind has been carried out at the Victoria Hospital, Glasgow, where a method of ventilation by propulsion, combined with purification or "washing" of the air, has been applied by Mr. William Key, C.E., of Glasgow. The New General Hospital, Birmingham, which is now in course of building, from designs by Mr. William Henman, a warm advocate of the artificial system, is to be supplied with a similar apparatus, and as the most recent and largest experiment of the kind in this country the results will be looked for with interest. The work will be carried out by Mr. Key. Steam is to be employed throughout the building for heating purposes, none of the piping, however, being fixed in any habitable portion of the hospital. The system adopted is known as Plenum. Air will be drawn in from four quarters through cleansing screens, constantly moistened and periodically flushed with water. In cold weather it will pass over large batteries of steam piping, and forced onward through ducts "of large sectional area to separate flues of proper dimensions to each room, ward, and corridor, at the foot of which are secondary steam heating coils, and a simple appliance by which both the temperature and volume of air can be regulated as required for each separate apartment." The cubical capacity of the building amounting to two million feet, provision is to be made for renewing the air ten times per hour, no less than twenty million cubic feet having thus to be cleansed, warmed, and propelled through the building every hour by night and day. There will be no fire-places.

We are very strongly of opinion that in England systems of this elaborate description are a mistake, and it is sincerely to be hoped that a spirit of emulation will not cause its application in England to any extent. In many of the most modern hospital buildings open fires, well-arranged systems of simple ventilation, and a certain amount of hot air or water heating in some cases, give results which could hardly be bettered. It is a noticeable fact that at King's College Hospital, "the home of Listerism," where atmospheric purity might be expected to reach its highest and best, that artificial ventilation is unknown, open fire-places and open windows are the order of the day (fires being kept burning all day and every day throughout the year in the wards), and more absolute sweetness and freshness in hospital precincts it would be hard to find.

It may therefore be taken that, so far, results go to prove that in England, at any rate, what is known as "natural ventilation" at present carries the day with the most reliable authorities on hospital construction, considered from the most practical point of view, and very remarkable progress must be made in the application of other systems in this country before their adoption can be sanctioned as desirable, or even justifiable, by those most competent to judge on the matter.

